# Impact of offspring endothelial function from *de novo* hypertensive disorders during pregnancy: An evidence-based review

**DOI:** 10.3389/fsurg.2022.967785

**Published:** 2022-11-07

**Authors:** Aslah Nabilah Abdull Sukor, Sheril June Ankasha, Azizah Ugusman, Amilia Aminuddin, Norfilza Mohd Mokhtar, Shahidee Zainal Abidin, Mohd Faizal Ahmad, Adila A. Hamid

**Affiliations:** ^1^ Department of Physiology, Faculty of Medicine, Universiti Kebangsaan Malaysia, Kuala Lumpur, Malaysia; ^2^ Faculty of Science and Marine Environment, Universiti Malaysia Terengganu, Kuala Nerus, Malaysia; ^3^ Department of Obstetrics and Gynaecology, Faculty of Medicine, Universiti Kebangsaan Malaysia, Kuala Lumpur, Malaysia

**Keywords:** hypertensive disorders of pregnancy, preeclampsia, endothelial function, offspring, cardiovascular disease

## Abstract

De novo hypertensive disorders of pregnancy (HDP) which consist of gestational hypertension and preeclampsia affect maternal and offspring morbidity and mortality, and potentially increase the risk of cardiovascular disease in the offspring. It is well known that *de novo* HDP causes various maternal complications, including cardiovascular diseases, placental abruption and liver and kidney failure. However, there are studies suggesting that offspring of pregnancies complicated by *de novo* HDP have an increased risk of long-term cardiovascular disease. The endothelium is an important regulator of vascular function, and its dysfunction is highly associated with the development of cardiovascular diseases. Hence, this review aimed to systematically identify articles related to the effect of *de novo* HDP on the endothelial function of the offspring. A computerized database search was conducted on PubMed, Scopus, and Medline from 1976 until 2022. A total of 685 articles were obtained. We identified another three additional articles through review articles and Google Scholar. Altogether, we used 13 articles for data extraction. All studies reported that endothelial function was impaired in the offspring of *de novo* HDP. This is most likely attributed to impaired vasodilation, subclinical atherosclerosis formation, inflammation, and dysregulated epigenetic regulation of endothelial functions.

## Introduction

Hypertensive disorders of pregnancy (HDP) are the most common medical disorder during pregnancy. HDP shows an increasing incidence rate globally with a total increase of 10.92% from 1990 to 2019 (1). The International Society for the Study of Hypertension in Pregnancy (ISSHP) classified HDP as chronic (pre-dating pregnancy or diagnosed prior to 20 weeks of pregnancy) or *de novo* (gestational hypertension or preeclampsia) (2, 3). Among all, preeclampsia (PE) was the most complex disorder, deteriorating rapidly with more than 70,000 maternal deaths and 500,000 fetal and neonatal deaths per year (2).

A previous study revealed that women with HDP have a higher tendency to have neonatal adverse outcomes than normotensive women (4). Alongside the complications of HDP to the mother, HDP also has short- and long-term adverse effects on the offspring. In the short term, neonates of mothers with HDP have a high risk of developing fetal hypoxia, premature birth, placental abruption, low birth weight, small for gestational age, respiratory distress syndrome, and death *in utero*. In the long term, neonates develop hypertension, arterial thickening, increased left ventricular wall thickness and reduced left ventricular end-diastolic volume (5).

Studies also found that HDP increases the risk of hypertension and cardiovascular disease (CVD) not only to the mother, but also to the offspring (6–8). The relevance of HDP on the offspring's health extends into adulthood. The HUNT study showed that as young adults, the offspring of women with HDP have higher CVD risk in terms of higher blood pressure (BP), body mass index (BMI) and waist circumference compared to the offspring of women with normal pregnancies (6).

HDP is considered a maternal and fetal endothelial disorder. The endothelium is a functional organ formed by a single layer of squamous endothelial cells that line all blood vessels. Endothelial cells play a role in vascular homeostasis and angiogenesis in response to injury or hypoxia. Studies have demonstrated that in preeclampsia, endothelial dysfunction occurs in the vascular endothelium of both mothers (9) and offspring (10). Preeclampsia is also the most common HDP that is associated with endothelial dysfunction (11).

Preeclampsia starts when placental perfusion is reduced by abnormal cytotrophoblast invasion of the spiral arteries (12). The imbalance between pro- and anti-angiogenic factors in preeclampsia is proposed to trigger the abnormal placental vascularization and the onset of preeclampsia (13). The dysfunctional placentation causes oxidative stress and increased resistance to placental blood flow. Subsequently, hypoperfusion, chronic placental ischemia (5) and endothelial dysfunction ensue (14). Endothelial dysfunction alters the capacity of endothelial cells to maintain homeostasis and leads to the development of CVD (15). Additionally, the premature offspring of women with preeclampsia had changes in their endothelial function from early life and a high risk to develop hypertension later (10).

The impact of *de novo* HDP on the offspring's endothelial function has just started to be valued. Most reviews highlight the effect of *de novo* HDP on maternal endothelial function, while the review on *de novo* HDP's effect on the offspring is limited, particularly the ones involving clinical studies. Therefore, this systematic review is aimed to evaluate the current database related to the outcome of *de novo* HDP, which includes gestational hypertension and preeclampsia, on endothelial function of human offspring *in utero*, at birth and long term.

## Method

### Search strategy

We identified the relevant studies on the effect of *de novo* HDP on offspring endothelial function using three electronic databases; PubMed, Scopus and Medline which were assessed between 1976 and 2022. The search strategy involved a combination (“AND”) of the following three sets of keywords: (1) Hypertension in pregnancy OR pre-eclampsia OR maternal hypertension OR gestational hypertension; (2) endothelial OR endothelial function OR endothelial dysfunction OR endothelial cell; and (3) neonate OR neonatal OR offspring OR fetal OR children. During the search, an asterisk (*) was used in Scopus as a truncation sign to broaden the search to include various word endings. We also searched the list of references of the articles selected for relevant citations.

### Inclusion and exclusion criteria

Studies that fulfill the following criteria were included: (i) studies that investigated the effects or association of *de novo* HDP (gestational hypertension or preeclampsia) on offspring endothelial function, (ii) human studies, (iii) studies published from 1976 to 2022, and (iv) articles published in English. The following studies were excluded: (i) studies that investigated the effects or association of chronic HDP on offspring endothelial function (ii) studies that associate CVD in neonates with congenital or other pathological changes unrelated to HDP (iii) review articles, meta-analyses, letters, newsletters, editorial, conference abstracts or case studies (iv) duplicated studies (v) animal studies and (iv) articles published in language other than English.

### Screening of articles for eligibility and data extraction

Firstly, articles that did not match the inclusion criteria based solely on their titles were excluded. Then, the abstracts of the remaining articles were screened to exclude articles that did not match the inclusion criteria. Finally, the full text of the remaining articles was read and assessed completely. At least two reviewers assessed the articles. Discussion between reviewers was done to resolve any issues. The data were extracted using a data collection form. The following data were extracted from the selected studies: (1) study population, (2) gestational/offspring age, (3) parameters measured, (4) findings, and (5) conclusion.

## Results

### Studies selected

**Figure 1:**
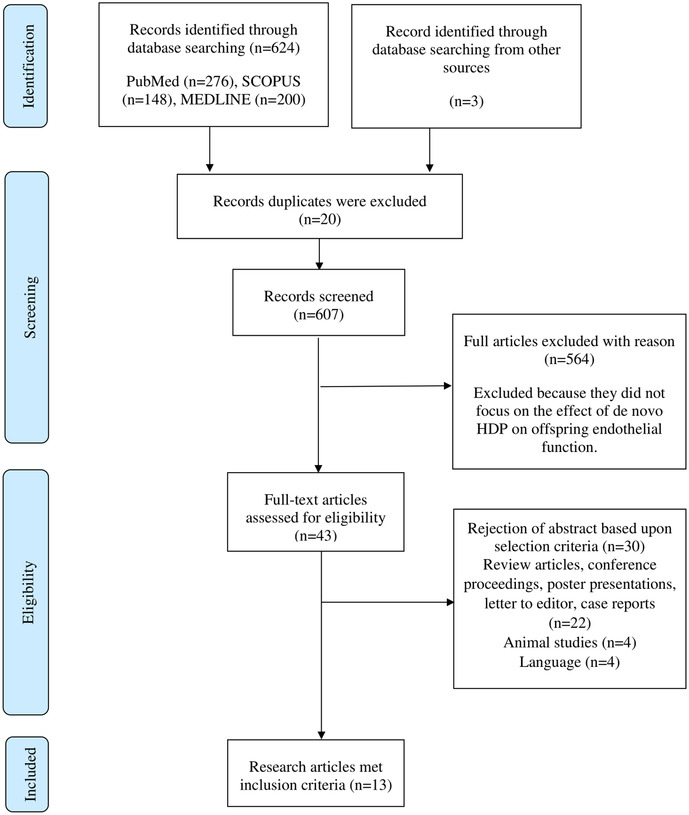
Summary of the steps involved in article selection process according to PRISMA guideline.

The initial literature search conducted in PubMed, Scopus and MEDLINE *via* EBSCOhost databases identified 685 potentially relevant articles. In the first phase, 643 articles were excluded as the articles were not related to the effect of *de novo* HDP on the offspring endothelial function based on the titles, abstracts, and keywords. Furthermore, 30 articles were excluded for several reasons: articles that are not original research, animal studies, articles not written in English, and duplicate articles. A hand-selected or snowball search was performed using Google Scholar, and we added another three relevant articles. After assessment by the reviewers, 13 articles were included in this review. The steps involved in the article selection process are shown in Figure 1. Based on the 13 included studies, the effects of *de novo* HDP on offspring endothelial function were classified into three major effects, namely, the impact on vasodilation and subclinical atherosclerosis (Table 1), inflammation (Table 2), and epigenetic regulation of endothelial functions (Table 3).

**Table 1:** Summary of the impact of *de novo* HDP on offspring vasodilation and subclinical atherosclerosis.

Refs	Study population	Gestational / Offspring age	Parameter (s)	Findings	Conclusion
(27)	- Preterm offspring of hypertensive pregnancies (*n* = 71) - Preterm offspring of normotensive pregnancies (*n* = 52) - Term offspring of normotensive pregnancies (*n* = 38)	18 months, 7 years, and 15 years old	- Peripheral and central SBP, DBP, MAP and PPBP - PWV, FMD, NMD and CIMT	- Offspring born preterm to either hypertensive or normotensive pregnancies displayed higher peripheral and central blood pressure compared to full-term born offspring. - Preterm offspring of normotensive pregnancies had greater arterial stiffness than offspring of hypertensive pregnancies. - Offspring of hypertensive pregnancies had higher CIMT and lower FMD.	Prematurity is associated with higher blood pressure in later life. Preterm offspring of hypertensive pregnancies have impaired endothelial function and greater subclinical atherosclerosis.
(17)	Healthy term newborns (*N* = 104)	First week of life	- Vasodilatation of the skin microvasculature in response to acetylcholine (endothelium-dependent vasodilator) and nitroprusside (endothelium-independent vasodilator)	- The maximum perfusion after administration of acetylcholine but not nitroprusside in the offspring was inversely associated with maternal hypertension.	Hypertension during pregnancy is inversely associated with endothelium-dependent vasodilatation of the offspring.
(16)	- Late onset gestational hypertension (*n* = 50) - Normotensive pregnancies (*n* = 50)	From 19th to 37th week of gestation	- Fetal aIMT, umbilical artery PI, fetal aorta PI and mean uterine arteries PI	- Fetuses of women affected by late gestational hypertension had higher fetal aIMT, umbilical artery PI, fetal aorta PI, and mean uterine arteries PI.	Gestational hypertension may predispose to impaired fetal cardiovascular development during intrauterine life.

Abbreviations: aIMT, Aortic intima-media thickness; CIMT, Carotid intima-media thickness; DBP, Diastolic blood pressure; FMD, Flow-mediated dilation; MAP, Mean arterial pressure; NMD, Nitrate-mediated dilatation; PI, Pulsatility index; PPBP, Pulse pressure blood pressure; PWV, Pulse wave velocity; SBP, Systolic blood pressure.

**Table 2:** Summary of the impact of *de novo* HDP on offspring endothelial inflammation and function.

(28)	- Offspring from PE pregnancies (*n* = 26) - Offspring from normal pregnancies (*n* = 17)	Five to eight years old	- Serum inflammatory biomarker (hsCRP) - RHI	- Lower RHI in children of PE with SGA pregnancies several years after birth. - No significant changes in the children's hsCRP levels. - No significant correlation between their RHI and hsCRP levels.	Children from PE pregnancies have reduced endothelial function.	
(18)	- HUVEC isolated from the offspring of women with mild PE (*n* = 7) - HUVEC isolated from the offspring of women with severe PE (*n* = 10) - HUVEC isolated from the offspring of normotensive women (*n* = 7)	At birth	- NO level in HUVEC	- HUVEC isolated from the umbilical cords of offspring from PE pregnancies displayed lower NO level. - Lower fetal endothelial NO level was associated with higher levels of maternal inflammatory markers (serum sE-selectin and VCAM-1).	Decreased NO level in fetal endothelium born from PE pregnancies was associated with high levels of maternal inflammatory markers.	
(19)	- Neonates of PE mothers (*n* = 65) - Neonates of normotensive mothers (*n* = 404)	At birth	- VCAM-1 in umbilical arteries - neonate coronary artery size	- Neonates of PE mothers had higher VCAM-1 expression in their umbilical arteries and larger coronary arteries at birth compared to neonates of normotensive women.	Coronary artery size is a useful severity index of neonatal endothelial inflammation due to PE.	

Abbreviations: hsCRP, High-sensitivity C-reactive protein; HUVEC, Human umbilical vein endothelial cells; NO, Nitric oxide; PE, Preeclampsia; RHI, Reactive hyperemia; sE-selectin, Soluble endothelial leukocyte adhesion molecule-1; SGA, Small gestational age; VCAM-1, Vascular cell adhesion molecule 1.

**Table 3:** Summary of the impact of *de novo* HDP on offspring epigenetic regulation of endothelial function.

Ref	Study population	Gestational / Offspring age	Parameter (s)	Findings	Conclusion
(20)	HUVEC isolated from the offspring of PE-complicated pregnancies (*n* = 3) and normotensive pregnancies (*n* = 3)	At birth between 36th – 39th week of gestation	- Differentially expressed miRNAs	- Sixteen differentially expressed-miRNAs including miR-29a/c-3p were downregulated in HUVEC isolated from PE pregnancies. - Knockdown of miR-29a/c-3p inhibited the FGF2-induced relative AKT1 phosphorylation in HUVEC - Knockdown of miR-29a/c-3p inhibited the VEGFA- and FGF2-induced endothelial cell migration in HUVEC.	PE dysregulates an array of miRNAs that may play important roles in fetal endothelial function. Downregulation of miR-29a/c-3p impairs the VEGFA- and FGF2-stimulated fetal endothelial cell migration.
(22)	HUVEC isolated from the offspring of PE-complicated pregnancies (*n* = 5-10) and normotensive pregnancies (*n* = 5-10)	At birth	- Endothelial transcriptome profile - Endothelial monolayer integrity, proliferation, and migration	- A total of 926 and 172 genes were dysregulated in HUVEC isolated from female and male offspring of PE pregnancies, respectively. - Many of the PE-dysregulated genes are associated with CVD and endothelial function. - TNF-α-, TGF*β*1-, FGF2-, and VEGFA-regulated gene networks were differentially disrupted in female and male HUVEC from PE pregnancies. - PE decreased endothelial monolayer integrity in response to TNFα in both female and male HUVEC. - PE decreased TGFβ1-strengthened monolayer integrity in female HUVEC, while it enhanced FGF2-strengthened monolayer integrity in male HUVEC. - PE promoted TNF-α-, TGFβ1-, and VEGFA-induced cell proliferation in female, but not in male HUVEC. - PE inhibited TNF-α-induced cell migration in female HUVEC but had an opposite effect on male HUVEC.	Preeclampsia dysregulates the fetal endothelial transcriptome and endothelial function in a sex-specific manner, with female offspring are more affected by PE than male offspring.
(21)	- Offspring and HUVEC isolated from the offspring of hypertensive pregnancies (*n* = 25) and normotensive pregnancies (*n* = 32)	- At birth between 37th – 39th week of gestation and 3 months postnatal	- Differentially expressed miRNAs - Dermal microvascular density	- Endothelial cells from neonates of hypertensive pregnancies had different miRNA profile associated with altered endothelial phenotype. - The most upregulated miRNA was miR-146a. - miR-146a overexpression led to impaired *in vitro* endothelial cell tubulogenesis. - Higher miR-146a level in the HUVEC of an offspring was associated with reduced microvascular density in the neonate at three months of life.	Offspring of hypertensive pregnancies have altered endothelial regulatory microRNA profile at birth, which is related to impaired endothelial cell behavior that predicts the patterns of microvascular development during the first three months of life.
(25)	Cord blood derived fetal ECFC from late onset PE (*n* = 12) and normotensive pregnancies (*n* = 12)	At birth	- Genomic methylation pattern	- Fetal ECFC from PE pregnancies showed a differential methylation pattern compared to uncomplicated pregnancies, with 954 genes in passage three and 1,719 genes in passage five showing differentially methylated CpG sites. - ECFC from PE pregnancies are differentially methylated in regions corresponding to a broad range of processes regulating cell metabolism, transcription, and cell cycle. - Regarding PE, the pathways involved might negatively impact the capacity for endothelial development/repair and trophoblast invasion.	- Fetal ECFC methylation status differs between PE and normotensive pregnancies. An epigenetically modified endothelial precursor may influence both normal morphogenesis and postnatal vascular repair capacity.
(26)	Cord blood-derived fetal EPC from PE (*n* = 12) and normotensive pregnancies (*n* = 9)	- At birth	- miRNA profile - Tube formation, chemotactic motility, and cell proliferation	- Cord blood-derived fetal EPC from PE pregnancies had different miRNA profile compared to healthy pregnancies. - The most statistically different miRNA was hsa-miR-1270 in cord blood fetal EPC from PE pregnancies, and the level was negatively correlated with the mRNA expression of its target gene, ANGPTL7 and TFRC. - Hsa-miR-1270 inhibition significantly reduced the tube formation and chemotactic motility but had no effect on the cell proliferation.	Cord blood-derived fetal EPC from pregnancies complicated with PE have different miRNA profile compared to healthy pregnancies, with hsa-miR-1,270 being one of the most differentially expressed miRNAs.
(23)	Cord blood-derived fetal EPC from PE (*n* = 14) and normotensive pregnancies (*n* = 10)	At birth	- Number of EPC in placental/fetal circulation - EPC proliferation, migration and vasculogenesis	Both circulating EPC and cultivated EPC were decreased compared with controls. Preeclampsia EPC were significantly impaired in their proliferation, migration and vasculogenesis capacities. Preeclampsia groups had higher cord blood level of soluble fms-like tyrosine kinase 1 (sFlt-1).	Preeclampsia reduces the number and function of fetal EPC which could be due to increased sFlt-1 levels.
(24)	Cord blood-derived fetal ECFC from PE (*n* = 15) and normotensive pregnancies (*n* = 35)	At birth	- ECFC levels - ECFC angiogenic function	- ECFC level in PE was lower than control. - There was no difference in the ECFC angiogenic function in terms of capillary network formation, endothelial colony formation, proliferative and migratory response.	ECFC level is lower in PE but there is no difference in the ECFC function.

Abbreviations: AKT1, AKT Serine/Threonine Kinase 1; ANGPTL7, Angiopoietin-related protein; ECFC, Endothelial colony forming cells; EPC, Endothelial progenitor cell; FGF2, Fibroblast growth factor 2; HUVEC, Human umbilical vein endothelial cells; miRNA, microRNA; mRNA, Mesengger RNA, PE, Preeclampsia; sFLT-1, Soluble fms-like tyrosine kinase-1; TFRC, Transferrin Receptor; TGFβ1, Transforming growth factor beta 1; TNFα, Tumor necrosis factor α; VEGFA, Vascular endothelial growth factor A.

### Study characteristics

The study design, including the age of the offspring and the study population differed in several ways. The age of the offspring involved in the studies varied greatly. There were a few different life stages, namely prenatal age (19th-37th week of gestation) (16), neonates (aged less than four weeks old) (17–26), infants (aged 2–12 months old) (21), toddlers (aged 1–3 years old) (27), children (aged 5–12 years old) (27, 28) and adolescent (aged 12–19 years old) (27). The study population also varied. Nine of the studies involved the offspring of mothers with preeclampsia (18–20, 22–26, 28), while the remaining four studies involved the offspring of mothers with gestational hypertension (16, 17, 21, 27).

### Impact of *de novo* HDP on offspring vasodilatation and subclinical atherosclerosis

A study by Touwslager et al. measured vasodilatation and perfusion of the offspring's skin microvasculature in response to acetylcholine (endothelium-dependent vasodilator) and nitroprusside (endothelium-independent vasodilator) using laser-Doppler device and iontophoresis. The maximum perfusion in the offspring's vasculature following acetylcholine administration, but not nitroprusside, was inversely associated with maternal HDP (17). In a 20-year follow-up study, offspring born preterm to either hypertensive or normotensive pregnancy had higher peripheral and central blood pressure than full-term born offspring (27). However, there were differences in their underlying vascular phenotype. Flow-mediated dilatation (FMD), which is the gold standard measurement for endothelial function, was lower in the preterm offspring of hypertensive pregnancies. The offspring also displayed a greater carotid intima-media thickness (IMT), a marker of subclinical atherosclerosis (27). In contrast, the preterm offspring of normotensive pregnancies had greater arterial stiffness than the offspring of hypertensive pregnancies (27). In another study, the fetus of mothers diagnosed with late gestational hypertension showed higher aortic IMT, umbilical artery pulsatility index (PI), fetal aorta PI and mean uterine arteries PI, which suggested subclinical atherosclerosis (16). In summary, the offspring from *de novo* HDP showed impaired vasodilatation and signs of subclinical atherosclerosis.

### Impact of *de novo* HDP on offspring endothelial inflammation and function

Children of women with a history of severe preeclampsia and small for gestational age pregnancies had impaired endothelial function as evidenced by reduced reactive hyperemia index (RHI) measured five to eight years after birth. However, there was no significant change in their circulating inflammatory biomarker level (high-sensitivity C-reactive protein; hsCRP), and there was no significant correlation between their RHI and hsCRP level (28). Meanwhile, human umbilical vein endothelial cells (HUVEC) isolated from the offspring of mothers with preeclampsia showed lower nitric oxide (NO) levels. NO is an important vasodilator and a marker of endothelial function. This reduction in fetal endothelial NO level was associated with higher levels of maternal inflammatory markers (serum soluble endothelial leukocyte adhesion molecule-1 (sE-selectin) and vascular cell adhesion molecule 1 (VCAM-1)) (18). Furthermore, offspring coronary artery size is a useful parameter to assess the severity of neonate's endothelial inflammation and dysfunction due to maternal preeclampsia. It was demonstrated that the neonates of mothers with preeclampsia had higher VCAM-1 expression in their umbilical arteries and larger coronary arteries, suggesting that coronary artery size is an indicator of neonatal endothelial inflammation (19). Taken together, the studies suggested that *de novo* HDP promotes inflammation which contributes to offspring's endothelial dysfunction.

### Impact of *de novo* HDP on offspring epigenetic regulation of endothelial function

Investigations on the epigenetic modifications of preeclampsia on offspring endothelial cells and endothelial progenitor cells (EPC) revealed an array of differentially expressed microRNAs (miRNAs or miRs), including hsa-miR-1270 (26), miR-29a/c-3p (20) and miR-146a (21). EPC are circulating cells that play an essential role in maintaining vascular function. Cord blood-derived fetal EPC from preeclampsia pregnancies had a different miRNA profile compared to healthy pregnancies. RNA sequencing analysis showed significant downregulation of hsa-miR-1270 in the EPC (26). The level of hsa-miR-1270 was negatively correlated with the mRNA expression of its target gene, angiopoietin-related protein 7 (ANGPTL7) and transferrin receptor (TFRC). ANGPTL7 and TFRC are important proteins involved in angiogenesis and cellular iron uptake, respectively. Furthermore, inhibition of hsa-miR-1270 decreased tube formation capacity and chemotactic motility of the EPC (26). Taken together, the results suggested that preeclampsia caused overexpression of hsa-miR-1270, which led to impaired angiogenic function of the offspring EPC. This corresponds with another study that showed preeclampsia reduced the number and function of fetal EPC, which could be due to an increase in the anti-angiogenic factor; soluble fms-like tyrosine kinase 1 (sFlt-1) (23).

Meanwhile, downregulation of miR-29a/c-3p was observed in HUVEC isolated from the offspring of preeclampsia pregnancies. MiR-29a/c-3p inhibition impaired vascular endothelial growth factor A (VEGFA) and fibroblast growth factor 2 (FGF-2)-induced endothelial cell migration (20). Additionally, upregulation of miR-146a was observed in HUVEC isolated from the offspring of hypertensive pregnancies. Overexpression of miR-146a led to impaired endothelial tubulogenesis*.* A higher miR-146a expression was also associated with reduced microvascular density in the offspring at three months of life, which might increase the risk of developing hypertension later (21). In short, the findings indicate that preeclampsia altered the expression of an array of miRNAs, which caused significant dysregulation of the offspring endothelial function.

Endothelial colony forming cells (ECFC), which is a proliferative subtype of EPC, was found to be reduced in the cord blood of offspring from preeclampsia pregnancies (24). Genomic methylation pattern of fetal ECFC from preeclampsia pregnancies also differed from normal pregnancies, with 954 genes in passage three and 1,719 genes in passage five showing differentially methylated CpG sites (25). This difference in genomic methylation pattern of ECFC in preeclampsia might negatively impact the capacity of offspring endothelial development and repair functions (25). In another study, endothelial transcriptome profiling revealed dysregulation of 926 and 172 genes in HUVEC isolated from the male and female neonate of preeclampsia pregnancies, respectively. Many of the dysregulated gene networks are associated with CVD and endothelial function, such as tumor necrosis factor α (TNF-α), transforming growth factor beta-1 (TGFβ1), FGF-2 and VEGFA (22). Further functional analysis showed a weakening of the endothelial monolayer integrity in HUVEC from female offspring in response to TNF-α. In the meantime, exposure to FGF-2 strengthened cell monolayer integrity in both male and female offspring endothelial cells. Preeclampsia also promoted TNF-α-, TGFβ1-, and VEGFA-induced proliferation of HUVEC from female offspring, but not in HUVEC from male offspring. In addition, preeclampsia inhibited TNF-α-induced migration of HUVEC of female offspring, with an opposite effect on HUVEC of male offspring (22). In summary, preeclampsia dysregulates the fetal endothelial transcriptome and endothelial function in a sex-specific manner, with female offspring more severely affected by preeclampsia than male offspring.

## Discussion

**Figure 2:**
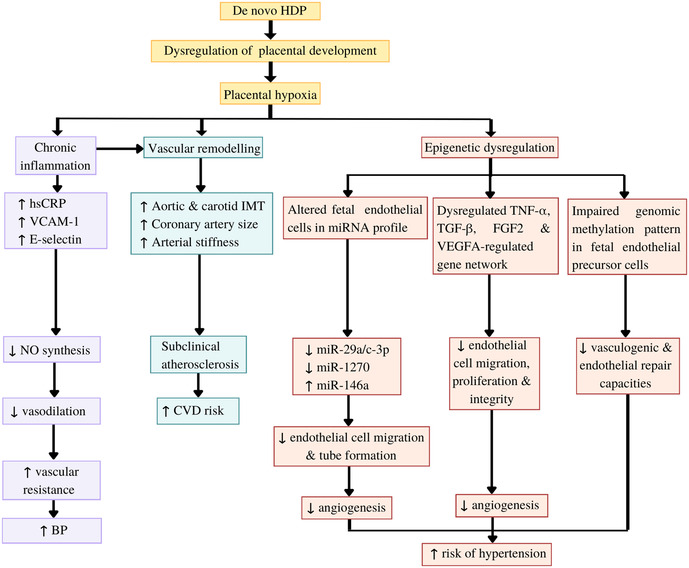
Potential mechanisms underlying the impact of *de novo* hypertensive disorders of pregnancy (HDP) on offspring endothelial function. HDP leads to dysregulation of placental development and placental hypoxia. Hypoxia induces chronic inflammation, which inhibits nitric oxide (NO) synthesis, resulting in impaired vasodilatation and increased blood pressure (BP). Both hypoxia and chronic inflammation cause vascular remodelling, which can be detected as subclinical atherosclerotic changes. Furthermore, hypoxia leads epigenetic dysregulation of the offspring endothelial function by causing alteration in the offspring endothelial cells and endothelial precursor cells microRNA (miRNA) profile, dysregulation of several gene networks related to endothelial functions, and impaired genomic methylation pattern in the endothelial precursor cells. These epigenetic dysregulations eventually caused impaired angiogenesis and endothelial repair capacities, which increase the risk of the offspring to develop hypertension (HPT) in later life. CVD: Cardiovascular diseases, FGF2: Fibroblast growth factor 2, hsCRP: high-sensitivity C-reactive protein, IMT: intima-media thickness, TGFβ1: Transforming growth factor beta-1, TNF*α*: tumour necrosis factor α, VCAM-1: vascular cell adhesion molecule 1, VEGFA: vascular endothelial growth factor A.

De novo HDP disrupted offspring endothelial function by causing impaired vasodilatation, subclinical atherosclerosis formation, inflammation, and epigenetic dysregulation of endothelial functions (Figure 2).

### HDP impairs vasodilatation and promotes subclinical atherosclerosis in the offspring

The endothelium is a thin membrane that lines the vascular network and plays an important role in vascular homeostasis. Maintaining the endothelium's structural and functional integrity is essential, particularly in balancing vasodilatation and vasoconstriction. Evidence suggests that offspring born to *de novo* HDP had impaired endothelium-dependent vasodilatation as measured by FMD (27), This result was in accordance with other studies done specifically in preeclampsia pregnancies (29, 30), indicating the possible increase in total peripheral resistance and blood pressure in the offspring. FMD is the gold standard method to measure endothelial function clinically. It is an ultrasound-based, non-invasive technique that measures endothelium-dependent vasodilatation in response to shear stress (31). However, further mechanistic studies are needed to fully understand the underlying mechanisms that contribute to the impaired vasodilatation in the offspring.

Microvascular structural changes can be seen in the offspring of mothers with HDP after birth due to heritable predisposition from the mothers or a reflection of intrauterine endothelial dysfunction (32). The aortic and carotid IMT are the markers of subclinical atherosclerosis, whereby IMT refers to the distance between the leading edge of the intimal interface and the leading edge of the media-adventitia interface at the outer part of the vessel (33). IMT is a well-established independent predictor of future CVD risk and previous studies have demonstrated a higher carotid IMT in children and adults with cardiovascular risk factors such as hypercholesterolemia (34), smoking (35) and family history of CVD (36). Fetuses of mothers with late gestational hypertension have higher aortic IMT detected at 29th -32nd weeks of gestation (16). This indicates vascular remodelling *in utero* (16). However, whether the increase in carotid IMT in HDP offspring is persistent or temporary is a matter of debate. A 20-year follow up study showed higher carotid IMT in the offspring of HDP (27). However, another study showed that the increase in offspring carotid IMT due to preeclampsia was attenuated at 18 months of life (37). Further studies are needed to address the underlying factors contributing to the difference. In short, *de novo* HDP leads to vascular dysfunction in the offspring by impairing the endothelium-dependent vasodilatation and inducing vascular remodeling such as the subclinical atherosclerosis formation.

### HDP promotes endothelial inflammation and dysfunction

In preeclampsia, chronic immune system activation leads to the release of proteins and factors by the placenta. This is part of the inflammatory response that promotes hypertension and proteinuria (38). The factors released include pro-inflammatory cytokines such as hsCRP and TNF-α, which have been reported to be elevated in mothers with preeclampsia (39). The human placenta secretes TNF-α under hypoxia-reoxygenation conditions *in vitro* (40), mimicking the fluctuation of oxygen levels observed in preeclampsia. TNF-α induces the expression of inflammatory cytokines such as interleukin (IL)-6, IL-8 and monocyte chemotactic protein (MCP-1) and the cellular adhesion molecules including VCAM-1, intercellular adhesion molecule 1 (ICAM-1), E-selectin and epithelial-cadherin (E-cadherin) (41). Furthermore, preeclampsia dysregulates the inducible nitric oxide synthase (iNOS) signaling, thus implicating higher inflammatory responses which potentially lead to severely impaired endothelial functions (22).

High maternal serum VCAM-1 and E-selectin levels in preeclampsia pregnancies was associated with 60%–70% decrease in NO level in the fetal endothelium (18). NO is a marker of endothelial function that acts as a vasodilator, thus reducing vascular resistance and blood pressure. Studies have shown that inhibition of NO synthesis results in reduced blood flow in human (18, 42, 43), vasoconstriction and increased blood pressure in animal offspring (44). This is further supported by another study that showed the RHI of preeclampsia offspring was reduced even at five to eight years after birth (28). RHI is a non-invasive method to measure endothelial function in which the response is partially mediated by endothelium-derived NO. The findings suggest that inflammatory response in preeclampsia inhibits NO synthesis, thus causing endothelial dysfunction and increased risk of hypertension.

### HDP causes epigenetic dysregulation of endothelial functions

Epigenetic plays an important role in the developmental origins of health and diseases, where its modifications would be potential mechanisms of the altered environment to be translated into disease development. MiRNAs are a class of noncoding RNAs that regulate essential cellular functions (45), including endothelial cell proliferation, migration, apoptosis, and angiogenesis (46). Previous studies demonstrated that stress conditions including *in utero* stress exposure and inflammation could alter endothelial miRNAs expression (20, 21, 47).

The altered miRNAs expression in *de novo* HDP impacts fetal endothelial functions through gene dysregulation of many signaling pathways. These include the estrogen signaling pathway (48), TGFβ signaling pathway (49), focal adhesion kinase pathway (50), phosphoinositide-3-kinase-protein kinase B (PI3K-Akt) signaling pathway (51), and also through impairment of VEGFA and FGF2 -stimulated angiogenesis (20). Moreover, the alteration of endothelial miRNAs in preeclampsia is not only associated with impaired endothelial cell function and behavior, but it also disrupts angiogenesis and microvascular development in infants as early as the first three months of life (20, 21). It was postulated that impaired angiogenesis *in utero* and early in life predisposes to hypertension development. However, on the brighter side, it is possible that microRNA modification and manipulation could restore the impaired angiogenesis, hence reducing the risk of the offspring to develop hypertension in later life (21).

An epigenetically modified endothelial precursor cell may influence both normal morphogenesis of endothelial cells *in utero* and postnatal vascular repair capacity, hence contributing to CVD risk in the offspring (25). EPC and ECFC are endothelial precursor cells originating from the bone marrow stem cells and human umbilical cord blood. Upon maturation, they become mature endothelial cells and release pro-angiogenic factors like VEGF and placental growth factor (PlGF), which enhance vasculogenesis and endothelial repair (52). Studies found that the numbers of EPC and ECFC in the offspring of preeclampsia mothers were reduced (23, 24), and the EPC were also found to be more senescent, consequently reducing their functional ability (53, 54). Altered number and function of fetal EPC in preeclampsia were associated with increased arterial stiffness (52), which is a risk factor for CVD. Besides, preeclampsia resulted in a different methylation pattern in fetal ECFC, with several differentially methylated regions identified in vascular-related genes (25). DNA methylation plays a pivotal role in regulating biological processes underlying CVD such as atherosclerosis, inflammation and hypertension (55–57). This suggests that epigenetic modifications in HDP may increase the risk of transgenerational vascular disease (58). Besides, these findings open the opportunity to introduce novel epigenetic targets for further experimental study.

### Strengths and limitations of the study

To the best of our knowledge, this is the first article that systematically reviewed current evidence related to the effect of *de novo* HDP on offspring endothelial function. The systematic literature search ensures all relevant articles were identified. Studies involving offspring from the prenatal period until adulthood were included, which enable us to understand the effects of *de novo* HDP on offspring endothelial function at different stages of life. However, the current review is not without its limitations. Only one of the studies involved the offspring of mothers with late onset gestational hypertension, while the rest of the studies did not specify the disease stage (i.e., early, or late). Therefore, comparison on the effect of early onset vs. late onset HDP on the offspring endothelial function could not be made. Comparing the effect of different stages of HDP on the offspring endothelial function is an interesting area to be explored. Furthermore, this review was only focused on human studies, while animal studies were excluded. Since animal studies are important tools for investigating how diseases in pregnancy can affect the offspring, further reviews that include animal models of hypertension in pregnancy are needed in the future.

## Conclusion

De novo HDP has a deleterious impact on offspring endothelial function. This is most likely attributed to impaired vasodilation, subclinical atherosclerosis formation, inflammation, and dysregulated epigenetic modification of endothelial functions. Endothelial dysfunction in the offspring of *de novo* HDP may contribute to their risk of developing CVD in later life. A cohort study involving this group of individuals is beneficial to establish the link between endothelial dysfunction in the offspring of HDP with CVD occurrence in adulthood for prevention and early intervention in the future.
